# Extracting a stroke phenotype risk factor from Veteran Health Administration clinical reports: an information content analysis

**DOI:** 10.1186/s13326-016-0065-1

**Published:** 2016-05-10

**Authors:** Danielle L. Mowery, Brian E. Chapman, Mike Conway, Brett R. South, Erin Madden, Salomeh Keyhani, Wendy W. Chapman

**Affiliations:** Department of Biomedical Informatics, University of Utah, Salt Lake City, UT USA; IDEAS Center, Veteran Affair Health Care System, Salt Lake City, UT USA; San Francisco Veteran Affair Health Care System, San Francisco, CA USA

**Keywords:** Natural language processing, Stroke, Phenotype, Information extraction

## Abstract

**Background:**

In the United States, 795,000 people suffer strokes each year; 10–15 % of these strokes can be attributed to stenosis caused by plaque in the carotid artery, a major stroke phenotype risk factor. Studies comparing treatments for the management of asymptomatic carotid stenosis are challenging for at least two reasons: 1) administrative billing codes (i.e., Current Procedural Terminology (CPT) codes) that identify carotid images do not denote which neurovascular arteries are affected and 2) the majority of the image reports are negative for carotid stenosis. Studies that rely on manual chart abstraction can be labor-intensive, expensive, and time-consuming. Natural Language Processing (NLP) can expedite the process of manual chart abstraction by automatically filtering reports with no/insignificant carotid stenosis findings and flagging reports with significant carotid stenosis findings; thus, potentially reducing effort, costs, and time.

**Methods:**

In this pilot study, we conducted an information content analysis of carotid stenosis mentions in terms of their report location (Sections), report formats (*structures*) and linguistic descriptions (**expressions**) from Veteran Health Administration free-text reports. We assessed an NLP algorithm, pyConText’s, ability to discern reports with significant carotid stenosis findings from reports with no/insignificant carotid stenosis findings given these three document composition factors for two report types: radiology (RAD) and text integration utility (TIU) notes.

**Results:**

We observed that most carotid mentions are recorded in *prose* using **categorical** expressions*,* within the Findings and Impression sections for RAD reports and within neither of these designated sections for TIU notes. For RAD reports, pyConText performed with high sensitivity (88 %), specificity (84 %), and negative predictive value (95 %) and reasonable positive predictive value (70 %). For TIU notes, pyConText performed with high specificity (87 %) and negative predictive value (92 %), reasonable sensitivity (73 %), and moderate positive predictive value (58 %). pyConText performed with the highest sensitivity processing the full report rather than the Findings or Impressions independently.

**Conclusion:**

We conclude that pyConText can reduce chart review efforts by filtering reports with no/insignificant carotid stenosis findings and flagging reports with significant carotid stenosis findings from the Veteran Health Administration electronic health record, and hence has utility for expediting a comparative effectiveness study of treatment strategies for stroke prevention.

## Background

In biomedicine, we define a disease or mutant phenotype experienced by an individual as observations caused by interactions between the environment and his/her genome that differ from the expected, “normal” wild type. Over the last several years, the biomedical community has begun to leverage informatics and electronic health record (EHR) data to define and identify phenotypes for genetic analyses using genome-wide (GWAS) and phenotype-wide (PheWAS) association studies [1, 2]. For instance, PheKB is a knowledgebase that contains phenotypes defined using EHR data and subsequently validated within one or more institutions. This catalogue of phenotypes was primarily generated by the Electronic Medical Records and Genomics (eMERGE) network, a United States (US) National Human Genome Research Institute-funded consortium, but is also supplemented by the informatics community at large (https://phekb.org/phenotypes) [3–5]. Similarly, the Strategic Health IT Research Program for secondary use of EHRs (SHARPn), funded by the US Office of the National Coordinator for Health Information Technology, aims to transform heterogeneous EHR data from various sites into a standardized form to support high-throughput phenotyping [6].

### Phenotyping with electronic health record data

Several phenotypes have been the foci of informatics studies including cancer, diabetes, heart failure, rheumatoid arthritis, drug side effects, cataract, pneumonia, asthma, peripheral artery disease, and hypertension [7]. EHRs provide a groundbreaking opportunity to define and identify these complex phenotypes leveraging data elements from the longitudinal patient record. Specifically, patient phenotypes are often inferred from both structured EHR data elements (e.g., administrative billing codes, vital signs, medications, laboratory values from data fields including dropdown lists and checkboxes) and unstructured EHR data elements (e.g., symptoms, signs, histories, and diagnoses within clinical notes including progress notes and discharge summaries). These heterogeneous data elements are then mapped to logical representations used to classify a patient into one or more phenotypes [8]. Outstanding challenges remain for next-generation phenotyping of EHR data including the need for approaches that address data complexity, inaccuracy, coverage, and biases [9].

### Natural language processing

Traditionally, International Classification of Disease (ICD-9) billing codes have been leveraged to identify phenotype risk factors with variable results. Inaccurate performance can result from poor granularity within code descriptions and documentation of risk factors in patient clinical texts [10, 11]. Natural language processing (NLP) may improve risk factor detection by identifying missed risk factor mentions (improving sensitivity) and filtering spurious risk factor mentions (improving positive predictive value) from these clinical texts. However, extracting risk factors associated with phenotypes from clinical texts can be challenging due to the usage of variable lexical expressions (e.g., “occlusion”, “reduced arterial diameters”), ambiguous abbreviations (PAD can stand for “peripheral artery disease” or “pain and distress”), spelling errors (“diabetes” misspelled as “diabeetes”), and telegraphic constructions (e.g., “PHx: HTN” means “past history of hypertension”) within clinical texts. Furthermore, multiple mentions of the same risk factor can be recorded within and across reports. This information might be integrated with structured data elements requiring logic to classify a patient with a phenotype. The success of an algorithm is often defined by performance metrics of sensitivity (or recall), positive predictive value (or precision), negative predictive value, and specificity by comparing the predicted phenotype from the system/algorithm against the coded phenotype from a domain expert [12].

### Extracting stroke risk factors using natural language processing

NLP has been applied and, at times, integrated with structured data to successfully identify several stroke risk factors such as peripheral artery disease [5, 13], diabetes [4, 14], heart failure [15], and hypertension [16] as part of large, coordinated research projects. Specifically, Savova et al. extended the Clinical Text Analysis and Knowledge Extraction System to extract and classify positive, negative, probable, and unknown mentions of peripheral artery disease (PAD) [13]. Kullo et al. then leveraged this system to encode case–control status, comorbidities, and cardiovascular risk factors from the EHR for a GWAS study of PAD cases and controls for the eMERGE project [5]. Wilke et al. applied the FreePharma system to extract medication histories and combine them with diagnoses and laboratory results to identify a diabetes mellitus cohort as part of the Marshfield Clinic Personalized Medicine Research Project (PMRP) [14]. Kho et al. extracted diagnoses, medications, and laboratory results leveraging NLP to encode variables from unstructured fields for various sites to identify type 2 diabetes cases and controls for a multi-institutional GWAS study also as part of the eMERGE project [4]. Garvin et al. extracted left ventricular ejection fraction as an indicator for heart failure using the Unstructured Information Management Architecture (UIMA) as part of a Translational Use Case Project and quality improvement project within the Veteran Affairs (VA) Consortium for Healthcare Informatics Research (CHIR) [15]. Finally, Thompson et al. translated the nine algorithms for phenotypes including hypertension developed from the eMERGE project into the Quality Data Model (QDM) to support EHR-based quality measures [16].

Although NLP has addressed many stroke-associated risk factors for genotype-phenotype and other studies, few studies have leveraged NLP to identify these risk factors specifically for stroke prevention research. Furthermore, to our knowledge, no NLP study has targeted significant carotid stenosis - a known risk factor for stroke. Our long-term goal is to develop a comprehensive stroke phenotyping framework that extracts predictors of stroke subtypes e.g., *ischemic or hemorrhagic* as well as their precise endotypes e.g., *ischemic stroke endotypes of cardiac embolism, large artery atherosclerosis, or lacunar infarction, other uncommon causes*, from the EHR powered by NLP. Our short-term goal is to develop an NLP algorithm for a National Institute of Health (NIH)-sponsored comparative effectiveness study of ischemic stroke prevention treatments that automatically filters carotid reports for patients exhibiting no/insignificant carotid stenosis of the internal or common carotid arteries from chart review. In this pilot study, we completed a qualitative and quantitative study of where and how mentions of carotid stenosis findings occur in radiology reports and how this affects an NLP algorithm’s performance.

## Methods

In this Institute Review Board (IRB or Ethics committee) and Veteran Affairs (VA) approved pilot study, we aimed to conduct an information content analysis of a major predictor of stroke, significant stenosis of the internal or common carotid arteries, for a sample of free-text reports from the Veteran Health Administration. Our goal is to automatically distinguish reports denoting one or more sides of significant stenosis (defined as greater than 50 %, moderate, or severe stenosis) from reports denoting no/insignificant stenosis (defined as negated, ruled out, mild, less than 50 % stenosis) from both of the internal or common carotid arteries. In this study, we conducted an information content analysis of carotid stenosis findings with respect to three aspects of document composition - location (Sections), format (*structures*), and descriptions (**expressions**). We assessed the performance of pyConText, an NLP algorithm, at automatically extracting and encoding stenosis findings given these three document constituents.

### Dataset

**Fig. 1:**
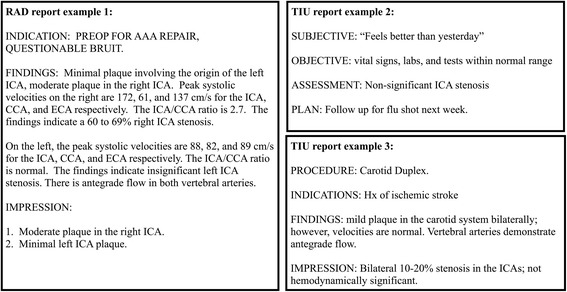
Sample texts by report type. Each text contains fictional, but realistic information

We selected all reports from the VA EHR for patients with an administratively documented carotid image procedure code (CPT code) restricted to those within −1 to +9 days of the procedure code date and that contained a carotid term (“carot”, “ica”, “lica”, “rica”, or “cca”). In our previous study, we leveraged 418 randomly sampled VA radiology reports for developing our NLP algorithm, pyConText, to identify mention-level stenosis findings [17]. We extended this previous study by randomly selecting a new set of reports to classify document-level stenosis based on identified mention-level carotid stenosis findings. This dataset consists of 598 radiology reports (RAD: mainly ultrasound reports) and 598 text integration utility notes (TIU: mainly progress notes, carotid duplex exams, and carotid triplex exams) (see Fig. 1). Because much of our algorithm development was completed during our previous study [17, 18] and the prevalence of stenosis positive reports is low, we chose a larger testing set for each report type. We also chose to maintain the natural distribution to give us a better sense of whether pyConText could correctly retain stenosis positive reports (high sensitivity) and to extrapolate the potential chart review savings from filtering stenosis negative reports (high negative predictive value). The dataset was randomly split into two sets: 200 development reports (100 RAD and 100 TIU notes) for algorithm knowledge base development [18] and 996 testing reports (498 RAD and 498 TIU notes) for information content analysis and algorithm evaluation. For the information content analysis, three research associates (domain experts) each independently and manually annotated the dataset for Sections, *structures*, and **expressions** as well as classified the report at the document-level as stenosis positive (if the report contained one or more mention of significant carotid stenosis) or stenosis negative (if the report contained only mentions of no/insignificant carotid stenosis). For the algorithm evaluation, the RAD reports were extracted from the VA EHR as two separate parts, Findings and Impressions. For the TIU reports, we parsed the Findings and Impressions using regular expressions written as a python script. We assessed pyConText’s performance when provided the Findings only, Impressions only, and the full report.

### Information content assessment

We aimed to characterize mentions of carotid stenosis findings according to Sections, *structures*, and **expression** types. Each report could have zero, one, or more relevant carotid stenosis findings recorded with zero, one, or more Sections, *structures*, and **expression** types.

#### Sections

RAD and TIU reports can be structured using canonical sections e.g., Indication, Findings, and Impression sections. We evaluated information content in the Findings (including Comments) versus Impressions (including Interpretations and Conclusions) sections [19].

#### Structures

VA notes can be generated using narrative or boilerplate templates in which the contents are saved as unstructured or semi-structured texts, respectively. For example, findings may be present in a variety of structures including: *prose*, *lists*, *tables*, *headings*, and *other* (Table 1). We evaluated information content according to these structure types [20].

**Table 1:** Structure types with example sentences

	Example sentence
*Prose*	“30–45 % stenosis in the right ICA.”
*List*	“1. Both ICAs are occluded.”
*Table*	“95 % RICA 50 % LICA 75 % LECA”
*Heading*	“Right: ICA: stenosis >70 %.”
*Other*	Any structures not listed above

#### Expressions

We have identified three types of expressions describing carotid stenosis findings: **category**, **range**, or **exact**. We characterized the information content according to these expression types [21] (Table 2).

**Table 2:** Expression types with example sentences

	Example sentence
Category	“severe stenosis”
Range	“stenosis ranging from 40 to 70 %”
Exact	“60 % stenosis”

### pyConText algorithm

pyConText is a regular expression-based and rule-based system that extends the NegEx [22] and ConText [23] algorithms. NLP developers can train pyConText to identify critical findings and their contexts by defining regular expressions for these targeted findings and their desired modifiers within its knowledge base, respectively [24]. These modifiers can be used to filter spurious finding mentions that would otherwise generate false positives if generating a cohort based on simple keyword search. For example, a negation modifier can reduce false positives by filtering denied findings e.g., “no carotid stenosis”. Furthermore, a severity modifier may reduce false positives by filtering insignificant findings e.g., “slight carotid stenosis”. In a previous study, pyConText identified pulmonary embolism from computed tomography pulmonary angiograms by filtering spurious mentions using modifiers of certainty, temporality, and quality with high sensitivity (98 %) and positive predictive value (83 %). The pyConText pipeline is composed of three main parts: *named entity recognition*, *assertion detection*, and *document-level classification*.

#### Named entity recognition and assertion detection

**Fig. 2:**
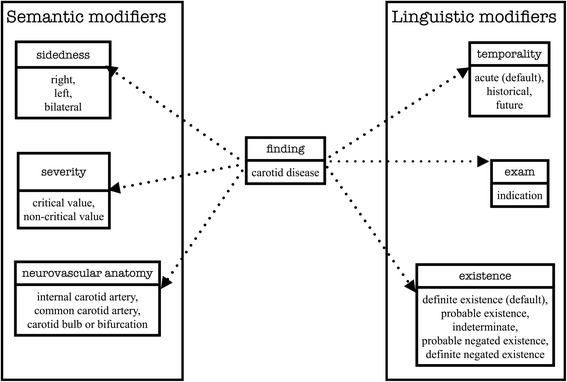
Schema representing findings as well as semantic and linguistic modifiers and their possible normalized value sets

**Fig. 3:**
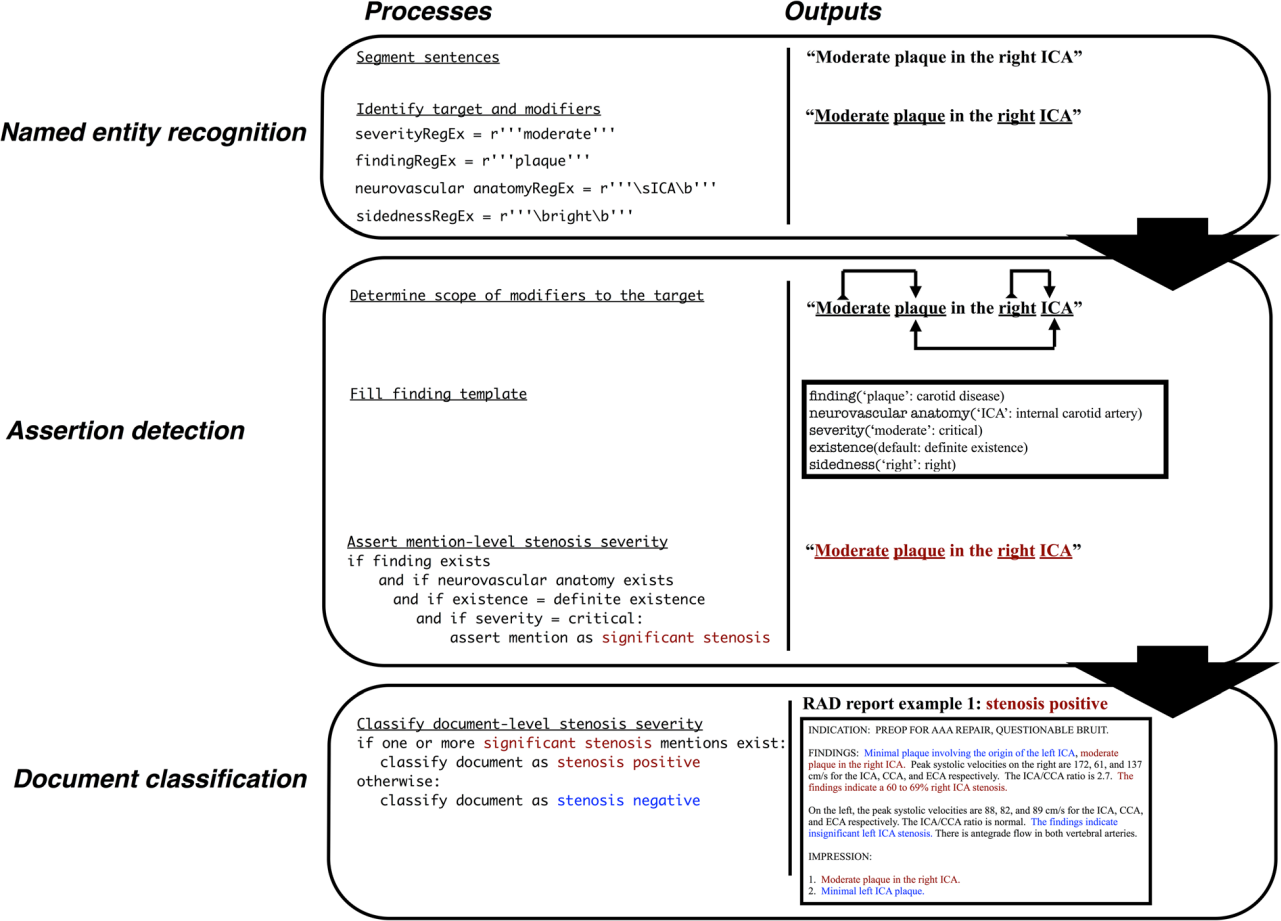
Illustration of pyConText’s pipeline encoding a sentence and classifying the document from Fig. 1 RAD report example 1. Some modifiers e.g., temporality and exam are not displayed for brevity. Blue mentions indicate templated mentions classified as no/insignificant stenosis; red mentions indicate templated mentions classified as significant stenosis

Specifically, we adapted pyConText’s knowledge base of findings and modifiers to filter no/insignificant carotid stenosis findings using regular expressions. These expressions contain “lexical variants” including synonyms, acronyms, abbreviations, and quantifications commonly documented in clinical text to represent carotid stenosis findings, semantic modifiers of severity, neurovascular anatomy, and sidedness, and linguistic modifiers of existence, temporality, and exam [25]. In Fig. 2, we provide the schema representing findings and each modifier as well as the possible normalized values. We represent these mentions and their normalized values using the following syntax: finding/modifier(‘lexical variant’: normalized value). For example, in Fig. 3, “Moderate plaque in the right ICA” is encoded as finding(‘plaque’: carotid disease), severity(‘Moderate’: critical value), neurovascular anatomy(‘ICA’: internal carotid artery), sidedness(‘right’: right), and existence(default: definite existence) using the knowledge base. pyConText leverages these normalized modifier values to determine whether a mention of a carotid finding(carotid disease) in the neurovascular anatomy(internal carotid artery, common carotid artery, carotid bulb or carotid bifurcation) represents no significant stenosis (stenosis with existence: definite negated existence), insignificant stenosis (stenosis with severity: non-critical value e.g., values less than 50 % stenosis), or significant stenosis (stenosis with severity: critical values e.g., values equal or greater than 50 % stenosis).

#### Document classification

**Fig. 4:**
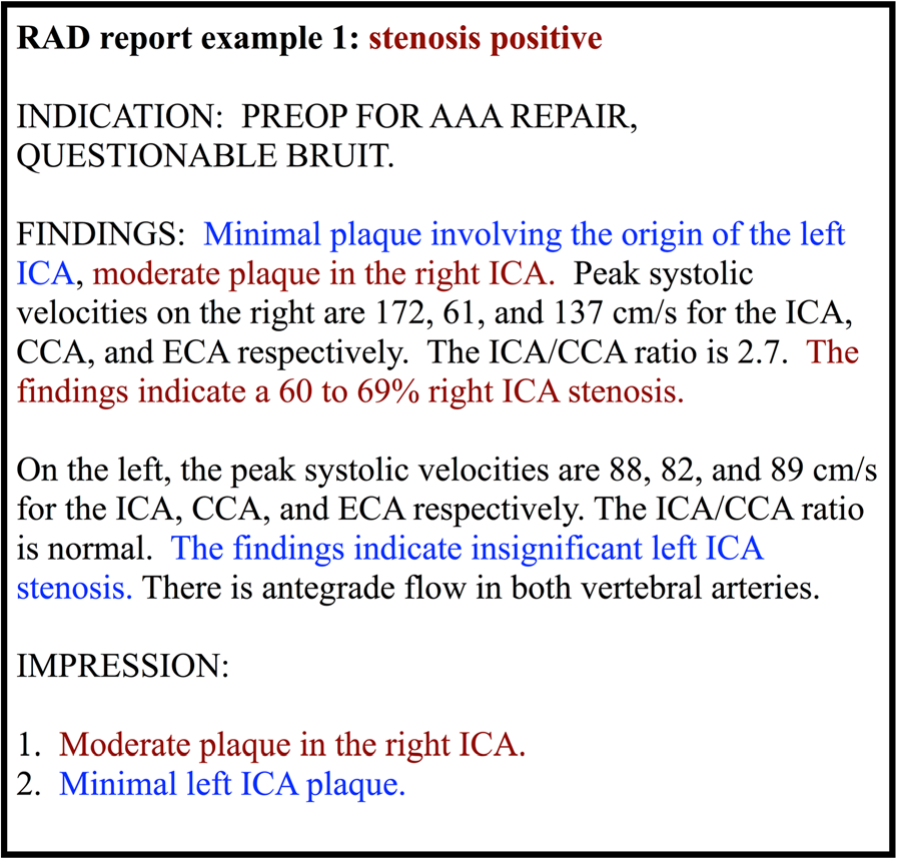
The resulting RAD report example 1 processed by pyConText from Fig. 3

For document-level classification, if either side or both sides of the internal or common carotid artery are determined to have significant stenosis, pyConText classifies the reports as stenosis positive; otherwise, it classifies it as stenosis negative. For RAD report example 1, in Fig. 3, the report would be classified as stenosis positive because two mentions of significant stenosis in the right internal carotid artery were identified. Figure 4 depicts RAD report example 1 fully processed by pyConText.

### pyConText evaluation

pyConText applies a simple processing approach of segmenting and tokenizing sentences to process reports. The algorithm does not make use of Sections and *structures*. Therefore, we quantified how frequently complex document composition - Sections, *structures*, and **expressions** - are utilized to report carotid stenosis findings to gauge whether document decomposition processing such as section or structure tagging is needed to accurately extract findings. We evaluated the frequency of errors by Sections, *structures*, and **expressions** by comparing the predicted report classifications by pyConText to those generated by our domain experts.

Specifically, we defined a true positive when a report is correctly classified by pyConText as stenosis positive and a true negative when a report is correctly classified by pyConText as stenosis negative. In contrast, we defined a false positive when a report is spuriously classified by pyConText as stenosis positive and a false negative when a report is spuriously classified by pyConText as stenosis negative [12]. We assessed pyConText’s performance by each Section and the full report using standard performance metrics of sensitivity, positive predictive value (PPV), specificity, and negative predictive value (NPV) as follows:

1. $$ sensitivity=\frac{true\  positive}{true\  positive+ false\  negative} $$
2. $$ positive\  predictive\  value=\frac{true\  positive}{true\  positive+ false\  positive} $$
3. $$ specificity=\frac{true\  negative}{true\  negative+ false\  positive} $$
4. $$ negative\  predictive\  value=\frac{true\  negative}{true\  negative+ false\  negative} $$

## Results

Our testing set was comprised of 498 radiology reports (RAD) ultrasounds and 498 TIU notes. At the document-level, for RAD reports, 353 (71 %) were stenosis negative and 145 (29 %) were stenosis positive; for TIU reports, 396 (80 %) were stenosis negative and 102 (20 %) were stenosis positive. The RAD training set distribution of 68 % stenosis negative and 32 % stenosis positive was comparable to the RAD testing set distribution. The TIU training set distribution of 87 % stenosis negative and 13 % stenosis positive reports differed slightly from the RAD testing set distribution.

### Information content assessment

Of the 498 RAD reports, we observed most carotid mentions occur within the Impressions (488), are recorded using *prose* (706), and are expressed as **categorical** expressions (713). Carotid mentions occurred often within both Findings and Impressions (359) (Table 3). In contrast, of the 498 TIU reports, we observed that most carotid mentions did not occur in either the Findings or Impressions (286). However, similarly to RAD reports, carotid mentions were recorded using *prose* (294), and were expressed as **categorical** expressions (344) (Table 3).

**Table 3:** According to report type, overall frequency of at least one carotid mention within sections, types of structures for all carotid mentions, and types of expressions for all carotid mentions

Information type	Information subtype	Report types	
		RAD	TIU
Sections			
	Findings Total	368	106
	Impressions Total	488	173
	Findings Only	9	39
	Impressions Only	129	106
	Both	359	67
	Neither/Not Applicable	1	286
*Structures*			
	*Prose*	706	294
	*List*	256	76
	*Table*	0	36
	*Heading*	46	152
	*Other*	2	6
Expressions			
	Category	713	344
	Range	254	314
	Exact	48	19

Findings Total = Findings only + Both; Impressions Total = Impressions only + Both. Neither = report has Findings and Impressions, but does not contain carotid mentions; Not Applicable = report does not have Findings and Impressions

For RAD reports, within Findings, most carotid mentions were recorded as *prose* (306) followed by *headings* (66); within Impressions, most carotid mentions were recorded as *prose* (352) followed by *lists* (127) (Table 4). In contrast, for TIU reports, within Findings, most carotid mentions were recorded as *headings* (43) followed by *tables* (33); as Impressions, most carotid mentions were recorded as *prose* (88) followed by *headings* (48) (Table 4).

**Table 4:** Structure type usage according to sections and report type

	*Prose*	*List*	*Table*	*Heading*	*Other*
RAD					
Findings	306	3	0	66	3
Impressions	352	127	0	22	0
TIU					
Findings	25	6	33	43	0
Impressions	88	21	13	48	0

For RAD reports, of the carotid mentions reported within both Finding and Impression (*n* = 359 reports; 379 paired mentions), there was repetition of structure types between sections (239 paired mentions, 63 %) (diagonals in Table 5). In cases where a different structure was used between sections (140 paired mentions, 37 %), the most frequent cases were Finding: *prose*/Impression: *list*, and Finding: *heading*/Impression: *prose* (discordants in Table 5). For TIU reports, of the carotid mentions reported within both Finding and Impression (*n* = 67 reports; 53 paired mentions), there was repetition of structure types between sections (22 paired mentions, 41 %) (diagonals in Table 5). In cases where a different structure was used between sections (31 paired mentions, 59 %), the most frequent cases were Finding: *table*/Impression: *prose* followed by Finding: *heading*/Impression: *list* and Finding: *heading*/Impression: *heading* (discordants in Table 5).

**Table 5:** Structure type usage between Findings (rows) and Impressions (columns) for repetitive mentions by report type

	*Prose*	*List*	*Table*	*Heading*	*Other*
RAD					
*Prose*	233 (61 %)	73 (19 %)	0 (0 %)	1 (<1 %)	0 (0 %)
*List*	1 (<1 %)	1 (<1 %)	0 (0 %)	0 (0 %)	0 (0 %)
*Table*	0 (0 %)	0 (0 %)	0 (0 %)	0 (0 %)	0 (0 %)
*Heading*	35 (9 %)	27 (7 %)	0 (0 %)	5 (1 %)	0 (0 %)
*Other*	2 (<1 %)	1 (<1 %)	0 (0 %)	0 (0 %)	0 (0 %)
TIU					
*Prose*	12 (23 %)	4 (7 %)	0 (0 %)	3 (6 %)	0 (0 %)
*List*	0 (0 %)	0 (0 %)	0 (0 %)	0 (0 %)	0 (0 %)
*Table*	15 (28 %)	0 (0 %)	1 (2 %)	0 (0 %)	0 (0 %)
*Heading*	0 (0 %)	9 (17 %)	0 (0 %)	9 (17 %)	0 (0 %)
*Other*	0 (0 %)	0 (0 %)	0 (0 %)	0 (0 %)	0 (0 %)

For RAD reports, both Findings and Impressions, most carotid mentions were expressed as **category** (330 and 381, respectively) followed by **range** (73 and 178, respectively) (Table 6). We observed similar trends for TIU reports: **category** (73 and 116, respectively) followed by **range** (59 and 110, respectively) (Table 6).

**Table 6:** Expression type usage by sections and report type

	Category	Range	Exact
RAD			
Findings	330	73	25
Impressions	381	178	23
TIU			
Findings	73	59	8
Impressions	116	110	5

For RAD reports, of the carotid mentions reported within both Findings and Impressions (*n* = 359 reports; 526 paired mentions), there was repetition of expression types between sections (345 paired mentions, 66 %) (diagonals in Table 7). In the cases where a different expression type was used between sections (181 paired mentions, 34 %), the most frequent cases were Finding: **category**/Impression: **range** and Finding: **range**/Impression: **category** (discordants in Table 7). For TIU reports, of the carotid finding mentions reported within both Findings and Impressions (*n* = 67 reports; 105 paired mentions), there was repetition of expression types between sections (45 paired mentions, 43 %) (diagonals in Table 7). Similar to RAD reports, in the cases where a different expression type was used between sections (60 paired mentions, 57 %), the most frequent cases were Finding: **category**/Impression: **range** and Finding: **range**/Impression: **category** (discordants in Table 7).

**Table 7:** Expression type usage between Findings (rows) and Impressions (columns) for repetitive mentions by report type

	Category	Range	Exact
RAD			
Category	278 (53 %)	108 (20 %)	14 (3 %)
Range	35 (7 %)	53 (10 %)	2 (<1 %)
Exact	16 (3 %)	6 (1 %)	14 (3 %)
TIU			
Category	30 (29 %)	23 (22 %)	1 (<1 %)
Range	26 (25 %)	13 (12 %)	3 (3 %)
Exact	3 (3 %)	4 (4 %)	2 (2 %)

### pyConText evaluation

For RAD reports, pyConText achieved the highest positive predictive value (80 %) and specificity (93 %) when provided Impressions only (Table 8). However, the algorithm performed with lower sensitivity (74 %) and negative predictive value (90 %) compared to performance when provided the full report performing with higher sensitivity (88 %) and negative predictive value (95 %). For TIU reports, we observed a similar trend. pyConText achieved the highest positive predictive value (76 %) and specificity (98 %) when provided Impressions only, but higher sensitivity (73 %) and negative predictive value (92 %) when provided the full report (Table 8).

**Table 8:** pyConText performance according to report type

	*Sensitivity*	*PPV*	*Specificity*	*NPV*
RAD				
Findings	57	67	88	83
Impressions	74	**80**	**93**	90
Full report	**88**	70	84	**95**
TIU				
Findings	60	55	88	89
Impressions	19	**76**	**98**	82
Full report	**73**	58	87	**92**

For each metric and report type, the highest metric value is bolded

For RAD reports, given the full report (including Findings and Impressions), pyConText generated 128 true and 56 false positive, and 297 true and 17 false negatives. The 73 reports were misclassified due to non-mutually exclusive errors of 96 *prose*, 42 *list*, 0 *table*, 12 *headings*, and 0 *other*. These non-mutually exclusive errors were the result of missed cues or erroneous scoping for 91** category**, 50 **range**, and 16 **exact** expressions. In terms of locality of errors, 53 mentions were in both section types, 1 mention was in Findings only, 19 mentions were in Impressions only, and 0 mentions were in neither section. For TIU reports, given the full report (including Findings and Impressions), pyConText generated 74 true and 53 false positive, and 343 true and 28 false negatives. The 81 reports were misclassified due to non-mutually exclusive errors of 58 *prose*, 10 *list*, 8 *table*, 50 *headings*, and 0 *others*. These non-mutually exclusive errors were the result of missed cues or erroneous scoping for 74 **category**, 85 **range**, and 2 **exact** expressions. In terms of locality of errors, 14 mentions were in both sections, five mentions were in Findings only, 21 mentions were in Impressions only, and 41 mentions were in neither section.

## Discussion

We conducted a pilot study evaluating information content of internal or common carotid finding mentions in terms of Section, *structure*, and **expression** usage. We also assessed pyConText’s performance given these three factors.

### Information content assessment

For RAD reports, most carotid mentions occurred in both Impressions and Findings with a substantial portion occurring in both sections. Overall mentions were recorded mainly as *prose* structure using **category** expressions. When carotid mentions were reported in Findings and Impressions, they were most often encoded in *prose*. For these cases, pyConText’s simple text processing can accurately extract most of these mentions. In many cases, carotid mentions are repeated between Finding and Impressions, mainly as *prose*. In the case of discordant structure usage, this redundancy can be a processing advantage. Specifically, one of the most frequent cases was Finding: *heading*/Impression: *prose.* Therefore, if given the full report, pyConText can still correctly extract carotid mentions from the Impressions when it incorrectly extracts mentions from the Findings due to more complex structures like *headings*. Most mentions were found in Impressions composed mainly using expressions of **category**. In cases of repetitive descriptions between Findings and Impressions, most are Finding: **category**/Impression: **category** and mentions with discordant structure usage were Finding: **category**/Impression: **range**. These observations suggest that most severity descriptions can be extracted leveraging lexical, qualitative (e.g., “severe”) regular expressions rather than quantitative (e.g., “70–99 %”) regular expressions.

For TIU reports, in contrast to RAD reports, most carotid mentions occurred in neither Findings nor Impressions, suggesting localized processing of reports for extracting carotid mentions would be suboptimal. In the few cases when carotid mentions were reported in Findings, they were most often *headings* followed by *table* structures. Similar to RAD reports, carotid mentions were reported in Impressions using *prose*, but also using *headings*, suggesting that complex document processing could be useful. Additionally, most mentions were found in Impressions composed mainly using expressions of **category** and exhibited the similar distributions of repetitive expression descriptions between Findings and Impressions.

For both RAD and TIU reports, we observed several mentions with two or more **expressions** or *structures.* For example, “55 % moderate ICA stenosis” contains two expressions: **exact** (55 %) and **category** (moderate).

### pyConText evaluation

We aimed to optimize the number of flagged positive cases for review (high sensitivity), while minimizing the loss of positive cases due to filtering (high negative predictive value); therefore, we conclude that pyConText performed best with the full report rather than with only the Finding or Impression sections. We hypothesize that providing pyConText with the full report resulted in the highest sensitivity because carotid mentions occurred with variable prevalence within Findings and Impressions (RAD) or within neither section type (TIU).

### Error analysis

A detailed error analysis of pyConText’s outputs revealed several areas of improvement to reduce false positives and negatives. For each error described, we provide an example and potential solution to boost performance within pyConText’s processing pipeline.

*Error 1:* For both RAD and TIU reports, some false positives were due to missing **category** or **range** expressions for semantic modifiers. For instance, in Example 1, although we had “small” as a non-critical value for severity and “moderate” as a critical value for severity, we did not have “small to moderate” in our knowledge base due to mixing of quality (small) and quantity (moderate) descriptors. In these cases, our domain experts used the lower bound (small) to classify the severity value and assert the carotid mention as insignificant stenosis. However, pyConText did not recognize this as a **range** expression and the upper bound (moderate) was incorrectly used to classify the severity value and assert the finding as significant stenosis.

*Example 1*. “small to moderate amount of calcified plague in the left carotid bulb”.

*Potential solution 1*: To improve assertion detection, we can add missed cues and expand upon existing regular expressions for the severity modifier. We could also add a rule that classifies ranges by the lowest bound for a severity value **range** by selecting the non-critical value over the critical value.

*Error 2:* In some cases, false positives were due to missing lexical variants for linguistic modifiers. In Example 2, we did not have a regular expression for “fails to demonstrate” for existence: definite negated existence; therefore, the algorithm classified the finding as significant stenosis.

*Example 2*. “examination of carotid arteries fails to demonstrate significant stenosis”.

*Potential solution 2*: To improve assertion detection, again, we can add missed cues and expand upon existing regular expressions to identify linguistic modifiers from the text.

*Error 3:* Sometimes, the expressions were correct, but spuriously attributed to flow velocities that were not used to assert stenosis findings as in Example 3.

*Example 3*. “diameter reduction.. cca with velocity of 82.

*Potential solution 3*: To improve assertion detection and scope, we could have created another modifier velocity to correctly scope the severity modifier and filter this mention from classification.

*Error 4:* Our results suggest that we achieved lower performance for TIU reports than RAD reports due to more frequent usage of complex document structures such *headings* and *tables* rather than less complex document structures of *prose* and *lists*. In Example 4, “ICA” was correctly attributed to “Left 40 % stenosis”, but not associated to “Right 30 % stenosis”.

*Example 4*. “ICA: Left 40 % stenosis.” “Right 30 % stenosis”.

*Potential solution 4*: To improve assertion detection and scope, we could boost pyConText’s performance by integrating outputs from a section tagger to identify mentions of neurovascular anatomy from *headings*/*subheadings* and associate them to all subsequent sentences within that section with relevant findings.

*Error 5:* In few examples, the algorithm generated a false negative due to its failure to identify co-referred findings of plaque. For Example 5, we observed two consecutive, long sentences. The first sentence contains a finding and neurovascular anatomy, but the second sentence contains its severity modifier. To link the severity in the second sentence to the finding and its neurovascular anatomy in the first sentence, we would need to resolve that the finding plaque in the second sentence co-refers to the finding plaque in the first sentence and merge their templates.

*Example 5*. “..calcified plaque in the left ica”…” “… data are consistent with between 50 and 80 % stenosis by plaque”.

*Potential solution 5*: To improve named entity recognition and assertion detection, we could handle co-reference, by identifying co-referring expressions and either merging or resolving conflicting values for each finding template.

*Error 6:* Not all failures resulted in a document misclassification. In Example 6, the finding is not given, but implied by the checkbox and associated modifiers of sidedness, neurovascular anatomy, and severity so pyConText did not extract a stenosis finding. However, if this statement represented a significant stenosis mention, a false negative would have resulted.

*Example 6*. “Left ICA [x]: 0–15 %”.

*Potential solution 6*: To improve named entity recognition and assertion detection, we could integrate outputs from document decomposition software [26] that readily identifies checkbox and question/answer constructs based on characters within the text. We could leverage these patterns to predict when and how these constructs should be used to extract assertions and correctly assert their scope when a finding is not explicitly mentioned.

*Error 7:* Similarly, although pyConText did not classify a finding mention in one sentence due to a missing modifier, it was able to identify and extract a finding mention from another sentence to correctly classify the report. In Example 7, pyConText does not find a neurovascular anatomy modifier for the second sentence, so it ignores it, but correctly classifies the report by correctly extracting information from the first sentence.

*Example 7*. “Right ICA occluded”… “1) occlusion on the right”.

*Potential solution 7*: To improve document classification, we could classify sentences without a neurovascular anatomy modifier, but this strategy would have caused a significant increase in the number of false positives when the mention represents an irrelevant neurovascular anatomy such as the external carotid artery, increasing the number of reports for chart review by abstractors.

*Error 8:* Finally, false positives could be attributed to a lack of topical context. In Example 8, the sentence does not contain an actual finding, but rather guidelines for classifying mentions as significant stenosis.

*Example 8*. “definitions: 70–99 % = significant stenosis”

*Potential solution 8*: To improve document classification, we could exclude extracted findings and assertions detected from all sentences that occur in the context of known guidelines e.g., documented NASCET legends by filtering these mention with a semantic modifier guidelines and regular expressions with guideline-associated keywords like “definitions”, “legend” or “NASCET”.

Although many of these solutions could prove useful, they may add significantly to pyConText’s processing time and complexity. For this study, it was only necessary to identify about 6,000 Veterans for cohort inclusion; therefore, we applied the system to the greater set of patient records based on these results. Because our goal is to retain as many stenosis positive cases as possible while filtering as many stenosis negative cases as possible, we provided pyConText the full report rather than only processing Impressions. To date, we have encoded over 150,000 RAD and 200,000 TIU reports. Given these results, we estimate that we have reduced the chart review task for study abstractors to about 85,000 (~25 %) of the possible reports. The manual review of this filtered set was completed in 4 months by three abstractors rather than 12 months without the NLP filtering.

### Limitations

Our study has a notable limitation. We only address reports from the VA EHR; therefore, pyConText’s performance may or may not generalize to reports from other institutions. However, if the reports contain similar Sections, *structures*, and **expressions**, we would expect similar results. We will evaluate pyConText’s generalizability on University of Utah Healthcare System reports for both genotype-phenotype association and stroke risk assessment studies in the near future.

### Future work

Although for this study, we developed a sensitive NLP algorithm to identify high risk patients for stroke to support a comparative effectiveness review study, we plan to extend our algorithm to extract additional stroke risk factors for precise stroke subtype phenotyping e.g., *ischemic and hemorrhagic stroke* subtypes and endotypes e.g., *ischemic stroke endotypes of cardiac embolism, large artery atherosclerosis, and lacunar infarction, other uncommon causes* for genotype-phenotype association studies. We are actively generating a pipeline with our knowledge base authoring system, Knowledge Author, to leverage existing vocabularies such as the Unified Medical Language System (UMLS) [27] and Radiology Lexicon (RadLex) as well as ontologies such as our Modifier Ontology to encode these stroke risk factors in a more streamlined manner [28, 29].

## Conclusions

We conclude that an information content analysis can provide important insights for algorithm development and evaluation including understanding information redundancy and challenges when processing clinical texts to identify stroke risk factors. Our study demonstrates that, in spite of these challenges, a simple NLP algorithm, can be leveraged to reduce chart review efforts by filtering reports with no/insignificant carotid stenosis findings and flagging reports with significant carotid stenosis findings from Veteran Health Administration clinical reports to support a comparative effectiveness study of stroke prevention strategies.

### Availability of the supporting data

The supporting annotated dataset contains protected health information and is stored in the Veteran Affairs Informatics and Computing Infrastructure (VINCI). It is not available to researchers outside of the Department of Veteran Affairs. However, pyConText is available through https://github.com/chapmanbe/pyConTextNLP. Additional study information and collaborative development for pyConText can be found at http://toolfinder.chpc.utah.edu/content/pycontext.

## Abbreviations

CPT: current procedural terminology
RAD: radiology
TIU: text integration utility
EHR: electronic health records
GWAS: genome-wide association studies
PheWAS: phenotype-wide association studies
ML: machine learning
NLP: natural language processing
eMERGE: electronic medical records and genomics
SHARPn: Strategic Health IT Research Program
PAD: peripheral artery disease
IRB: Institute Review Board
VA: veteran affairs
CHIR: consortium for healthcare informatics research
PPV: positive predictive value
NPV: negative predictive value
UMLS: unified medical language system
RadLex: radiology lexicon
VINCI: veteran affairs informatics and computing infrastructure
PMRP: personalized medicine research project
UIMA: unstructured information management architecture
QDM: quality data model
NIH: National Institute of Health
